# Thermal Reaction: The Spread of Bisphenol S via Paper Products

**DOI:** 10.1289/ehp.121-a76

**Published:** 2013-03-01

**Authors:** Lindsey Konkel

**Affiliations:** Lindsey Konkel is a Worcester, MA–based journalist who reports on science, health, and the environment. She writes frequently for *Environmental Health News* and *The Daily Climate*.

In January 2013 county executive Steve Bellone of Suffolk, New York, made history when he signed the “Safer Sales Slip Act,” a first-in-the-nation ban of thermal receipt paper coated with bisphenol A (BPA).^1,2^ BPA, used as a developer in thermal papers, has been linked in some animal studies to adverse reproductive and metabolic effects, while epidemiologic evidence shows an association with thyroid effects.^3^ But the safety of structurally similar bisphenol S (BPS), a widely used BPA replacement, has itself come under question, with one recent study indicating the compound disrupts cell signaling at extremely low doses.^4^ Although scientists are just beginning to analyze the safety of BPS, studies suggest this compound is already pervasive in the environment,^5^ in paper products,^6^ and in the human body.^7^

In a draft report released in July 2012, Design for the Environment, a research partnership program of the U.S. Environmental Protection Agency, assessed 19 chemical alternatives to BPA in receipt paper, including BPS.^8^ The preliminary report found no clear winners as far as human health and environmental end points go.

“It appears that BPS and BPA are equally problematic” in terms of toxicity, says Kurunthachalam Kannan, a research scientist at the New York State Department of Health in Albany and senior author of three recent BPS studies.

Kannan’s group reported in 2012 that 81% of 315 urine samples from men and women in the United States and seven Asian countries contained BPS.^7^ The highest average concentrations were found in urine samples from Japan, followed by samples from the United States. Japan banned BPA-coated thermal receipt papers in 2001.^5,6,7^ Appleton, a major U.S. manufacturer of thermal receipt paper, switched from BPA to BPS in 2006 due to growing concern about the safety of BPA, says corporate communications manager Bill Van Den Brandt.

**Figure:**
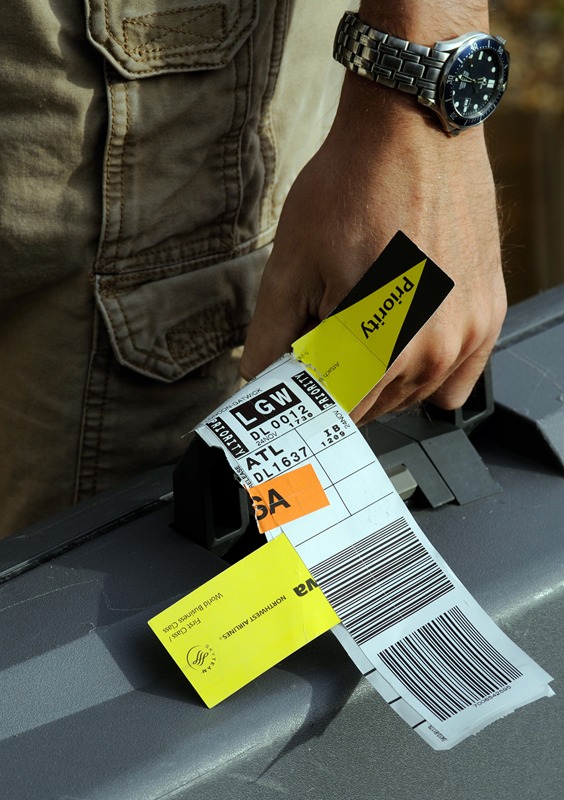
Relatively high levels of BPS have been measured in thermal paper receipts, tickets, boarding passes, and luggage tags. © Peter Titmuss/Alamy

In another 2012 study Kannan and colleagues analyzed 16 types of paper and estimated that receipt paper accounted for more than 88% of human BPS exposure from handling papers.^6^ They also found relatively high BPS concentrations in tickets, mailing envelopes, and airline boarding passes and luggage tags, which also are printed using thermal processes.^6^ Papers coated with BPS may contain up to 40% more compound than comparable papers coated with BPA, perhaps because BPS is a weaker developer than BPA, according to Frederick vom Saal, a biologist at the University of Missouri-Columbia’s Endocrine Disruptors Group.

Although thermal papers may contain a lot of BPA and BPS, it remains unclear how much of the chemicals makes it into the human body, according to Laura Vandenberg, a postdoctoral fellow at Tufts University. Studies estimate between 10% and 60% of BPA from receipt paper is absorbed through the skin.^9^ In one small sample of pregnant women, cashiers had higher urinary BPA levels than women in other occupations.^10^ There are no similar data for BPS. “Future studies for both chemicals must focus on how blood concentrations of the chemicals change before and after touching receipt paper,” Vandenberg says.

The detection of small quantities of BPS in paper products that are often made with recycled content—such as napkins, flyers, and magazines—suggests that BPS, like BPA, is transferred from thermal paper that has been recycled.^6^ According to a 2008 European Commission report, as much as 10% of thermal paper produced is recycled as scrap before it ever enters commerce, and an estimated 30% of used thermal paper also is recycled.^11^ The amount of BPA released during recycling can vary widely, depending on the processes used. Comparable information is unavailable for BPS, which may be more persistent in the environment than BPA.^12^

There are no studies testing how readily the body absorbs BPS or BPA from recycled paper goods. But Vandenberg points out that the chemicals in these products are mixed in with the paper rather than coated on top, so there may be less opportunity for dermal absorption. Still, she says, “Some recycled paper products, like facial and toilet tissues, come into contact with our mucous membranes and sometimes our food. We don’t want that paper to have chemicals that mimic hormones.”

But of greater concern than recycled products, says vom Saal, is the immediate consequence of touching thermal paper coated with free BPA or BPS. “You will spread that chemical onto everything you touch until you wash your hands,” he says.
